# Analysis of the costs of teleconsultation for the treatment of diabetes mellitus in the SUS

**DOI:** 10.11606/s1518-8787.2024058005433

**Published:** 2024-04-12

**Authors:** Frederica Valle de Queiroz Padilha, Daniela Laranja Gomes Rodrigues, Gisele Silvestre Belber, Marcos Aurélio Maeyama, Lígia Spinel, Ana Paula Neves Marques Pinho, Alessandra Vitti, Mariana Selbach Otero, Greta Barriquel Pompermaier, Tanise Balvedi Damas, Haliton Oliveira

**Affiliations:** I Hospital Alemão Oswaldo Cruz Departamento de Sustentabilidade e Responsabilidade Social São Paulo SP Brasil Hospital Alemão Oswaldo Cruz. Departamento de Sustentabilidade e Responsabilidade Social. São Paulo, SP, Brasil; II Instituto de Estudos de Políticas de Saúde São Paulo SP Brasil Instituto de Estudos de Políticas de Saúde. São Paulo, SP, Brasil; III Universidade Federal de Santa Catarina Núcleo de Telessaúde Florianópolis SC Brasil Universidade Federal de Santa Catarina. Núcleo de Telessaúde. Florianópolis, SC, Brasil; IV Secretaria Municipal de Saúde Joinville SC Brasil Secretaria Municipal de Saúde. Joinville, SC, Brasil

**Keywords:** Costs and Cost Analysis, Remote Consultation, Diabetes Mellitus, Unified Health System

## Abstract

**OBJECTIVE:**

To present the results of a cost analysis of remote consultations (teleconsultations) compared to in-person consultations for patients with type 2 diabetes, in the Brazilian public healthcare system (SUS) in the city of Joinville, Santa Catarina (SC). In addition to the costs from the local manager’s perspective, the article also presents estimates from the patient’s perspective, based on the transportation costs associated with each type of consultation.

**METHOD:**

Data were collected from 246 consultations, both remote and in-person, between 2021 and 2023, in the context of a randomized clinical trial on the impact of teleconsultation carried out in the city of Joinville, SC. Teleconsultations were carried out at Primary Health Units (PHU) and in-person consultations at the Specialized Health Center. The consultation costs were calculate by the method time and activity-based costing (TDABC), and for the estimate of transportation costs data was collected directly from the research participants . The mean costs and time required to carry out each type of consultation in different scenarios and perspectives were analyzed and compared descriptively.

**RESULTS:**

Considering only the local SUS manager’s perspective, the costs for carrying out a teleconsultation were 4.5% higher than for an in-person consultation. However, when considering the transportation costs associated with each patient, the estimated value of the in-person consultation becomes 7.7% higher and, in the case of consultations in other municipalities, 15% higher than the teleconsultation.

**CONCLUSION:**

The results demonstrate that the incorporation of teleconsultation within the SUS can bring economic advantages depending on the perspective and scenario considered, in addition to being a strategy with the potential to increase access to specialized care in the public network.

## INTRODUCTION

Restrictions on access to healthcare by the population during the SARS-CoV-2 pandemic accelerated the demand and adoption of new technologies, especially telemedicine, not only for the care of people affected by the coronavirus, but also for those with other acute and/or chronic conditions who were in a situation of social isolation^1^. The use of this strategy has shown potential to increase accessibility to health services, reduce travel time and opportunity costs related to the seeking care^2^. Furthermore, it can increase the diversity of treatments to which an individual has access, especially those who live in remote areas^6^. However, this type of technology requires appropriate infrastructure, trained staff, and revised care processes to support the service, as well as effective change management strategies to support both clinical and administrative teams and patients.

Teleconsultation is already an authorized practice with specific legislation in several countries, and in some—such as Australia, Japan, and Finland^7^since the 1990s. Several studies demonstrate equivalence between teleconsultation and in-person consultation in the treatment of patients with chronic diseases and other illnesses, within specific contexts and conditions^8,9^.

Regarding costs, the way the service is implemented and the perspective used (from the health system, the patient, or society) are decisive in the cost-effectiveness results of this technology^10^, and the savings generated by reducing or eliminating the need for patients and doctors to travel have proven be the main economic advantage of teleconsultations over in-person consultations^4,5,10,11^.

In Brazil, the Federal Council of Medicine (CFM) published a resolution in 2018 regulating remote medical care, which was soon revoked and subject to adjustments^12^. In 2020, with the advent of the COVID-19 pandemic, the Ministry of Health, on an exceptional and temporary basis, regulated teleconsultation actions to provide medical care without the risk of spreading the disease. After the end of the Public Health Emergency, a new resolution was published^13^ defining and guiding medical professionals in the use of this technology for patient care and follow-up.

With recent regulations, studies on the topic at a national level are still incipient. It is in this context, of lack of evidence on the effectiveness of teleconsultation in the country, that the Teleconsultation Project of the Hospital Alemão Oswaldo Cruz (HAOC), a randomized clinical trial (RCT)^14^ within the scope of the Program to Support the Institutional Development of the SUS (Proadi-SUS), is situated. Its objective is to test the hypothesis of non-inferiority of teleconsultation compared to in-person consultation in patients with type 2 diabetes, as well as to analyze and compare the costs of the two methods in the SUS; the latter objective is the focus of this article.

Based in the city of Joinville, SC, the study works with a specialized teleconsultation model in which the patient goes to the primary health care unit (PHU), in a safe environment and with the support of a technical health professional, to interact by video call with a specialist doctor located in a Specialized Health Center (Polyclinic). In-person consultations take place directly at the Polyclinic.

The objective of this article is to present the results of an analysis of the costs of both types of consultation, remote and in-person, using the micro-costing methodology Time-Driven Activity-Based Costing (TDABC)^15^. We work on the hypothesis that the two types of consultation modes are non-inferior, a hypothesis of the RCT supported in other studies^8,9^.

This analysis was carried out from the perspective of costs for the health system (SUS) and for the patient, considering different arrangements for travel costs. By comparing the costs of the different types of consultation, we intend to provide evidence to support subsequent analyses of the productive capacity of the PHU and their ability to absorb teleconsultation within the SUS financing model.

## METHODS

### Study Design

This is a micro-costing analysis that provides information on the cost of a specialized consultation in remote and in-person modalities, with an endocrinologist, for the treatment of type 2 diabetes, considering the non-inferiority of the teleconsultation mode. The analyses come from patients recruited in a randomized, phase II, pragmatic and non-inferiority clinical trial, whose protocol has already been published previously^14^.

The methodology used to calculate costs from the perspective of the health system is Time-Driven Activity-Based Costing (TDABC)^15^, which was adopted because it allows the identification of the cost at the unit level of the service within the expected efficiency conditions, and it also serves as a comparison metric. This methodology has already been applied to measure costs in various health services^17^and even in telemedicine services nationwide^21^.

TDABC starts from mapping the activities involved in the process of interest and the resources involved in each of them. It then estimates the *capacity cost* rate of the resources, determined by the equation:


$$T_{0}=C_{of}/C_{p}$$

In which: *Tc* = capacity cost rate; *Ccf* = cost of the capacity provided; *Cp* = practical capacity of the resource provided.

Ccf is the cost of a given resource to carry out the activity and *Cp* is the number of minutes spent carrying out this activity. Otherwise, this rate can be interpreted as the cost per unit of time (minute) of resources involved in a given activity^15^. In the TDABC model, practical capacity is considered to be 80% to 85% of theoretical capacity, assuming that approximately 20% of working time is usually spent in intervals and 15% of equipment usage time is spent on maintenance and repairs^16^.

For the calculation from the patient’s perspective, the costs related to travel (transport costs) to the Polyclinic and to the PHU were estimated. Data was collected through a questionnaire filled out by patients following the consultation, with questions about the type of transport used and travel time to each of the locations (PHU and Polyclinic).

Finally, to extrapolate the analysis to other municipalities, data was used regarding the source of financing for the transport of patients according to SAS Ordinance No. 055/1999 (TFD – Treatment Outside the Home)^22^—which guarantees transportation and accommodation for treatment. According to data in the TFD table (Ordinance No. 2,488/2007), for cases in which the municipality does not provide transportation, the patient receives an allowance of R$8.40 for food without an overnight stay and R$4.75 for every 50km of land travel.

### Sample

The sample consisted of 246 patients, who were included in the randomized study according to pre-established inclusion criteria^14^. The 246 consultations were carried out between January 2021 and January 2023 and refer to the patients’ first consultation with specialist doctors, with 123 consultations for each modality.

Table 1 shows the descriptive statistics regarding the participants’ profile in terms of gender and age group.

**Table 1:** Descriptive statistics on the participants’ profile in the two types of consultation.

Consultation type	% Male	% Female	Mean age (years)	Standard deviation (age)
In-person	39	61	60.12	9.83
Teleconsultation	42	58	60.23	11.04

Source: Data from the Teleconsultation Project

### Data Source

The calculation of the mean salary/month of doctors, nursing technicians and the administrative team of the PHU and Polyclinic was made based on data available on the Transparency Portal of the Municipality of Joinville^23^.

Data relating to the cost of materials and consumable items were made available by the Joinville Municipal Health Department for three PHU and used as a reference for the cost of materials and consumables for the other units and for the Polyclinic.

For the costs of the equipment necessary for teleconsultation (computer, videoconference platform, digital signature), the amounts spent within the scope of the Teleconsultation Project were used, considering a useful life of 5 years (20% depreciation rate) and a intertemporal discount of 4.5% per year (2020 average IPCA-IBGE inflation index).

The time related to pre-consultation procedures (patient registration and screening) was collected by the project’s field researcher and the duration of the consultations was recorded by the responsible endocrinologists, considering the beginning and end of the consultation.

To analyze transportation costs, values were assigned to bus fares, fuel usage and taxi or app vehicles travels in 2020 (the year for which all calculations were made), based on the data collected in the questionnaire answered by patients after their consultation. A total of 135 patients completed the entire questionnaire, all of whom provided data on their travel to the nearest PHU and 93 to the Polyclinic (42 participants had never been to the institution).

### Data analysis

The mapping of activities that are part of the two types of consultation was carried out from the flowchart provided in the Teleconsultation Project^14^. As shown in the Chart, the activities of the two types of consultation and the professionals involved are the same, with the exception of testing equipment and its use, in the case of remote consultation.

**Chart:** Activities involved in the in-person consultation and teleconsultation process.

Teleconsultation		In-person consultation	
Subactivity	Responsible	Subactivity	Responsible
Reception and registration of patients	PHU Administrative	Reception of patients	Administrative Polyclinic
Carrying out preliminary exams (vital signs)	PHU nursing technician	Carrying out preliminary exams (vital signs)	Polyclinic nursing technician
Equipment testing	PHU nursing technician		
Teleconsultation	Specialist doctor	Consultation	Specialist doctor

To calculate transportation costs, data on the average travel time to each health unit and the relative cost of each means of transportation were considered, weighted by the proportion of patients who reported using them.

The analyses focused on a description of the costs and duration of the different steps of the consultations, with information on mean costs and percentage comparisons.

The evaluation of the difference between the average duration of each type of consultation was carried out using the Student’s *t*-test in the R software.

## RESULTS

The practical capacity considered for nursing technicians and administrative staff was 176 hours, or 10,560 minutes (80% of 220 hours/month). In the case of endocrinologists, the practical capacity considered was 96 hours, or 5,760 minutes (80% of 120 hours/month).

For the category of materials and consumable items, 85% of their theoretical capacity was considered, according to the TDABC model, resulting in 36,720 minutes/month of practical capacity and, for the equipment needed for teleconsultations, the practical capacity considered corresponds to the doctors’ workload (96 hours/month), based on the assumption that this equipment will be used exclusively for teleconsultations. The value for a PHU is considered here.

From this data, the capacity cost rate, or cost per minute, was calculated, as shown in Table 2, where it can be seen that, in the case of Joinville, the mean cost for administrative staff at the PHU is higher than that observed at the Polyclinic. In the case of nursing technicians, there is no significant difference.

**Table 2:** Capacity cost rate: cost per minute of resources.

Activities	Hours/month	Minutes/month	Mean salary/cost/month	Standard deviation (R$)	Capacity Cost Rate (R$)
Nursing technician	176	10,560	5,346.48	1,425.06	0.51
Mean-PHU					
Nursing technician	176	10,560	5,408.42	1,871.02	0.51
Mean-Polyclinic					
Administrative staff	176	10,560	4,546.22	1,756.42	0.43
Mean-PHU					
Administrative staff	176	10,560	3,060.78	927.11	0.29
Mean-Polyclinic					
Doctors	96	5,760	10,584.00	2,049.14	1.84
Materials and consumables	612	36,720	2,547.31	NA	0.07
Teleconsultation equipment	96	5,760	288.10	NA	0.05

Source: data from the Joinville Transparency Portal and data from the Teleconsultation Project.

NA: not applicable.

In the second stage, the duration required to complete each activity was collected. In in-person consultations, the mean duration of patient registration was 9.6 minutes and the time for carrying out preliminary examinations was 5.3 minutes and, in teleconsultation, 10.5 and 4.7, respectively.

On average, consultations last 42.8 minutes, with a teleconsultation being less than 1 minute (48 seconds) shorter than an in-person consultation. The standard deviation was also lower in the remote mode: 8.8 minutes compared to 9.6 minutes in in-person mode.

Considering a significance level of 95%, we do not reject the null hypothesis that the mean durations of the described stages are statistically equal. Thus, the duration considered for estimating costs using the TDABC model for both types of consultation was: 5 minutes for registering patients, 10 minutes for carrying out preliminary exams and 42.8 minutes for carrying out consultations.

### Cost from the SUS Perspective: Calculation of Accounting Cost

After obtaining the costs of the resources used throughout the process, these were associated with the respective activities and their mean duration, resulting in an accounting cost of R$ 92.4 for a teleconsultation, which is 4.5% higher than that for an in-person consultation, which was R$ 88.4 (Table 3). It was also shown that the step of carrying out the consultation accounts for more than 90% of the costs, because of the value of the doctors’ salaries in relation to other costs.

**Table 3:** Cost per activity in different consultation types.

Subactivity	Responsible	Teleconsultation					Face-to-face consultation				
		Cost/min (R$)		Time (min)	Cost (R$)	% of the cost of each step	Cost/min (R$)		Time (min)	Cost (R$)	% of the cost of each step
		Salaries (R$)	Structure and materials				Salaries (R$)	Structure and materials			
Patient registration	Administrative staff	0.43	R$ 0.07	10	5	5	0.29	R$ 0.07	10	3.6	4
Preliminary exams (vital signs)	Nursing technician	0.51	R$ 0.07	5	2.9	3	0.51	R$ 0.07	5	2.9	3
Equipment testing	Nursing technician	0.51	R$ 0.11	1	0.62	1					
Teleconsultation	Specialist doctor	1.84	R$ 0.12	42.8	83.9	91	1.84	R$ 0.07	42.8	81.9	93
Total					92.41	100				88.42	100

The additional value of teleconsultation in relation to in-person consultation is the result of the cost of the equipment to carry out the consultation and the higher salary of administrative staff at the PHU.

In this scenario, the difference in costs between the two modalities is quite sensitive to variations in the duration of the consultation. For example, for a situation in which, on average, the duration of a teleconsultation is one minute shorter than that of an in-person consultation, the accounting cost of the two modalities would be the same.

This estimate of unit cost per consultation assumes that the entire period of practical capacity of the PHU is used to carry out teleconsultations, or an average service of 135 consultations/month per PHU.

### The Patient Perspective and Travel Costs

In addition to the accounting cost, we examined the patients’ perspective by incorporating cost estimates related to their travel in each type of consultation in the analyses.

Table 4 presents the mean travel time and cost for each location, considering round-trip routes.

**Table 4:** Mean travel cost to the PHU and Polyclinic (round trip).

Displacement	Boa Vista Polyclinic						PHU						Diff. mean travel time Polyclinic-PHU (min)
	n	%	Mean journey time (min)	Standard deviation (min)	Travel cost (R$)	Weighted cost (R$)	n	%	Mean journey time (min)	Standard deviation (min)	Travel cost (R$)	Weighted cost (R$)	
Car	55	59	48	37	12	7.1	70	52	24.6	27.8	6.2	3.2	23.4
Taxi/app	17	18	34.4	38.7	44	8	10	7	20.4	31.5	26.1	1.9	14
Bus	18	19	67.5	40	9.5	1.8	4	3	20.5	38.9	9.5	0.3	47
Bicycle/walk	3	3	25	35.3	0	0	51	38	22.5	33.2	0	0	2.5
Total	93	100	48.5			17	135	100	23.4		41.8	5.4	25.2

In Joinville, a medium-sized city with approximately 600,000 inhabitants, the mean cost of transportation for a consultation at the Polyclinic (R$ 17.00) is more than three times higher than for a consultation at the neighborhood PHU (R$ 5.40).

Regarding travel time, the average travel time to the Polyclinic is 25 minutes longer than to the nearest PHU. The biggest difference is in bus travel, where the reported travel time to the Polyclinic is 47 minutes longer. In fact, only 3% of the sample declared that they used to use this means of transport to go to their neighborhood PHU, where, due to its proximity, 38% said that they used to go on foot or by bicycle.

Adding the cost from the patient’s perspective to the calculation of the accounting cost, we obtain a scenario in which the cost of an in-person consultation is 7.7% higher than that of a teleconsultation:


$$\begin{array}{cc} & {\text{ TELEC }}_{\text{oot }}=92.4+5.4=97.8\\ & IN-PERSONC_{\text{ost }}=88.4+17=105.4 \end{array}$$

### Estimation of Transportation Costs for Patients from other Municipalities

In this estimation we established a scenario in which the diabetes treatment via the SUS begins in primary care in the user’s city of origin and is referred to other locations that have complementary specialized care services. In these cases, it is the obligation of the municipality of origin to transport or bear the costs of transporting patients and even companions for treatment away from home. In the case of specialized endocrinology consultations, the municipality of Joinville is a reference for six other municipalities (Araquari, Barra do Sul, Garuva, Itapoá, São Francisco do Sul) in the region^a^. In other words, all these municipalities have to bear the costs of transport and food for patients who travel to Joinville for a specialized consultation with an endocrinologist.

Considering that the cost of carrying out a teleconsultation in the PHUs of these municipalities is equivalent to that calculated and using the TFD values for a municipality 50 km away from Joinville, in the case of an in-person consultation there is an additional R$17.90, which includes transportation and food for the patient, without a companion.


$$\begin{array}{cc} & {\text{ TELEC }}_{\text{oot }=}92.4\\ & IN-PERSONC_{\text{oot }}=88.4+17.9=106.3 \end{array}$$

In this scenario, considering only the accounting cost from the perspective of the SUS, the in-person consultation would be 15% more expensive than the teleconsultation carried out at a local PHU.

Still in this case, we can analyze the perspective of different managers: that of the patient’s municipality of residence and that of the municipality where the consultation took place, in this case, Joinville. Within the SUS financing system for municipal funds, the cost of the consultation would be in Joinville, so it makes sense to compare, from the perspective of the manager of the patient’s municipality of residence, the transportation costs with the costs of carrying out the teleconsultation at the local PHU. In this case, based on the values shown in table 3, the cost for the local manager would be R$ 10.66, that is, 40% lower than the transport cost calculated from the TFD data.

## DISCUSSION

In this analysis, the costs of teleconsultation were slightly higher than those of the in-person category, but were quite sensitive to variations in parameters, such as the duration of the consultation. When the patient’s perspective is considered, or in a scenario in which the patient has to travel to another city to carry out the consultation, the remote modality presents a lower cost. The opportunity cost related to the time “saved” by patients in the remote consultation modality was not considered, but in large urban centers and intercity travel it can be quite relevant.

In the case of consultations held in other municipalities, the management and cost of transportation vary greatly, but the amount can be significant. In a study^24^carried out in the municipality of Santa Maria (in the state of Rio Grande do Sul), the mean cost per patient transported was R$ 32.94, plus an additional R$ 2.40 per kilometer traveled. Another study^25^ in Victor Meireles (in the state of Santa Catarina), with just 5,000 inhabitants, found a mean monthly expenditure of R$37,835.16 on patient transport.

Other economic aspects could be impacted by the implementation of teleconsultation, such as user absenteeism from appointments and exams, which generates a large waste of resources. In Brazil, studies point to absenteeism rates of over 25% in specialized care^26,27^, which tend to be higher than those recorded in primary care^27,28^. Considering that the distance to the place of care is inversely related to the absenteeism rate^29^, the possibility of providing specialized care closer to home, or even in patients’ home, could provide a more efficient use of resources by possibly reducing missed appointments.

Evidence from other countries also highlights the economic potential of teleconsultation. In a systematic review^30^of studies conducted in high-income countries using quality-adjusted life years, the results indicate that telemedicine is cost-effective and, in the context of low-income countries, another study with data from more than 25,000 teleconsultations suggests considerable economic benefits and great potential for telemedicine to improve the treatment of chronic diseases in low-resource areas^31^.

In this sense, the availability and quality of resources can be an obstacle to the implementation of teleconsultation and the realization of the benefits associated with it. In the model discussed here, where teleconsultations are carried out in PHU, the units already had items such as connection and room to carry out the teleconsultation, but a recent study^32^points out that most PHU do not have a minimum structure for carrying out a teleconsultation, with regional and population size inequalities and greater needs in the regions that could most benefit from such a service, like the North and Northeast.

In addition to infrastructure, the low remuneration for consultations and the inequality in the distribution of doctors across the territory are some of the factors that contribute to the provision of specialized care being one of the major bottlenecks in the system^33,34^, where telemedicine has the potential to serve as a a mitigating factor, if combined with the provision of services in a hierarchical and regionalized manner, ensuring an adequate economy of scale. Our results contribute to the body of evidence on the topic at a national level, but present some limitations. The estimated absolute values do not correspond exactly to the values of a “real” consultation, as they were calculated within the scope of a specific study. However, the figures relating to the difference between the cost of the two types of consultation, together with other aspects discussed here, offer estimates of the economic impact of implementing this technology, which should also be examined in relation to the population’s level of need and the subsequent effects on access to healthcare^3^, especially in a public and universal system.

As it is a regional study (Joinville/state of Santa Catarina), personnel remuneration values may differ from other Brazilian locations. Furthermore, the external validity of the data, considering the implementation of teleconsultation in scenarios other than endocrinology, and more specifically diabetes, is low.

Further studies complementing the theme of this evaluation, carried out in a multicenter manner and with other medical specialties, can add greater strength to this analysis and identify the potential and limits of telemedicine in Brazil.

## CONCLUSION

Our findings demonstrated that the costs for carrying out a teleconsultation in a PHU were 4.5% higher than for an in-person consultation carried out in a specialized center. The durations of the two types of consultation are statistically the same, however, with the doctors’ remuneration accounting for more than 90% of the costs, the estimates are quite sensitive to variations in the length of the consultation: all other parameters being held constant, If the teleconsultation lasted one minute less, the cost of both types of consultation would be the same.

When considering the transportation costs for the patient and for cases in which the municipality is responsible for the travel costs, the value of the in-person consultation becomes, respectively, 7.7% and 15% higher than that of the remote modality.

## Footnotes

a Information provided by the Joinville Municipal Health Department.
