# Supplementary material for: Analysis of the costs of teleconsultation for the treatment of diabetes mellitus in the SUS
Source: Rev Saude Publica. 2024 Apr 12;58:15. doi: 10.11606/s1518-8787.2024058005433 (PMC11037897; doi:10.11606/s1518-8787.2024058005433)
Supplement: Translation

# Translation: Análise dos custos da teleconsulta para tratamento de diabetes mellitus no SUS

**Authors:** Frederica Valle de Queiroz Padilha, Daniela Laranja Gomes Rodrigues, Gisele Silvestre Belber, Marcos Aurélio Maeyama, Lígia Spinel, Ana Paula Neves Marques Pinho, Alessandra Vitti, Mariana Selbach Otero, Greta Barriquel Pompermaier, Tanise Balvedi Damas, Haliton Oliveira

**Affiliations:** I Brasil Hospital Alemão Oswaldo Cruz. Departamento de Sustentabilidade e Responsabilidade Social. São Paulo, SP, Brasil; II Brasil Instituto de Estudos de Políticas de Saúde. São Paulo, SP, Brasil; III Brasil Universidade Federal de Santa Catarina. Núcleo de Telessaúde. Florianópolis, SC, Brasil; IV Brasil Secretaria Municipal de Saúde. Joinville, SC, Brasil

**Keywords:** Custos e Análise de Custo, Consulta Remota, Diabetes Mellitus, Sistema Único de Saúde

## Abstract

**OBJETIVO:**

Apresentar os resultados da análise de custos para a modalidade de consulta remota (teleconsulta) em comparação à consulta presencial, em pacientes com diabetes do tipo 2, no Sistema Único de Saúde (SUS) brasileiro, na cidade Joinville, Santa Catarina. Para além dos custos, sob a perspectiva do gestor local, o artigo também apresenta estimativas sob perspectiva do paciente, a partir dos custos de transporte associados a cada modalidade de consulta.

**MÉTODO:**

Foram coletados dados de 246 consultas, remotas e presenciais, entre 2021 e 2023, no contexto de um ensaio clínico randomizado sobre o impacto da teleconsulta realizado na cidade de Joinville, SC. As teleconsultas foram realizadas em Unidades Básicas de Saúde (UBS) e as consultas presenciais no Centro de Saúde Especializada. Para o cálculo dos custos, foi utilizado o método de custos baseado em tempo e atividade (TDABC) e, para o cálculo dos custos relativos aos transportes dos pacientes, foram coletados dados diretamente com os participantes da pesquisa. Foram analisados e comparados descritivamente os custos médios e o tempo de realização de cada modalidade de consulta em diferentes cenários e perspectivas.

**RESULTADOS:**

Considerando apenas a perspectiva do gestor local do SUS, os custos para a realização da teleconsulta se mostraram 4,5% maiores do que para uma consulta presencial. Contudo, quando considerados os custos de transporte associados a cada paciente, o valor estimado da consulta presencial passa a ser 7,7% maior e, no caso de consultas em outros municípios, 15% maior do que a teleconsulta.

**CONCLUSÃO:**

Os resultados demonstram que a incorporação da teleconsulta dentro do SUS pode trazer vantagens econômicas, a depender da perspectiva e do cenário considerado, além de ser uma estratégia com potencial para aumentar o acesso à atenção especializada na rede pública.

## INTRODUÇÃO

As restrições de acesso à saúde pela população, durante a pandemia do SARS-CoV-2, aceleraram a demanda e a adoção de novas tecnologias, em especial a telemedicina, não só para o cuidado de pessoas acometidas pelo coronavírus, como também de pessoas portadoras de outras afecções agudas e/ou crônicas, que se encontravam em situação de isolamento social^1^. O uso desta estratégia tem mostrado potencial para aumentar a acessibilidade aos serviços de saúde, reduzir o tempo de viagens e os custos de oportunidade relacionados ao processo de obtenção de cuidados^2^. Ademais, pode aumentar a diversidade de tratamentos aos quais um indivíduo tem acesso, sobretudo aqueles que vivem em áreas remotas^6^. Contudo, este tipo de tecnologia requer infraestrutura apropriada, equipe treinada e processos assistenciais revisados para dar suporte ao serviço, além de estratégias eficazes de gerenciamento de mudanças para apoiar tanto as equipes clínicas e administrativas quanto os pacientes.

A teleconsulta já é uma prática autorizada e com legislação específica em diversos países, sendo que, em alguns – como Austrália, Japão e Finlândia^7^–, desde os anos 90. Diversos estudos demonstram equivalência entre a teleconsulta e a consulta presencial no tratamento de pacientes com doenças crônicas e outras enfermidades, dentro de contextos e condicionalidades específicas^8,9^.

Com relação aos custos, a forma de implantação do serviço e a perspectiva utilizada (do sistema de saúde, do paciente ou da sociedade) são determinantes nos resultados relativos à custo-efetividade dessa tecnologia^10^e a economia gerada pela diminuição ou supressão do deslocamento de pacientes e médicos tem se mostrado a principal vantagem econômica da teleconsulta em relação à consulta presencial^4,5,10,11^.

No Brasil, o Conselho Federal de Medicina (CFM) publicou, em 2018, uma resolução regulamentando o atendimento médico a distância, logo revogada e sujeita a ajustes^12^. Em 2020, com o advento da pandemia de covid-19, o Ministério da Saúde, em caráter excepcional e temporário, regulamentou as ações de teleconsulta para prestar atendimento médico sem risco de propagação da doença. Após o fim da situação de Emergência de Saúde Pública uma nova resolução foi publicada^13^, definindo e orientando os profissionais médicos no uso desta tecnologia para o atendimento e acompanhamento de pacientes.

Com uma regulamentação recente, ainda são incipientes os estudos sobre o tema em âmbito nacional. É neste contexto, de carências de evidências sobre a efetividade da teleconsulta no país, que se insere o Projeto Teleconsulta do Hospital Alemão Oswaldo Cruz (HAOC), um ensaio clínico randomizado (ECR)^14^ no âmbito do Programa de Apoio ao Desenvolvimento Institucional do Sistema Único de Saúde (Proadi-SUS), que tem como objetivo testar a hipótese de não-inferioridade da teleconsulta em comparação à consulta presencial em pacientes com diabetes do tipo 2, assim como analisar e comparar os custos das duas modalidades no Sistema Único de Saúde (SUS); este segundo objetivo é foco deste artigo.

Com base na cidade de Joinville, SC, o estudo trabalha com um modelo de teleconsulta especializada em que o paciente vai à unidade básica de saúde (UBS), em ambiente seguro e com apoio de um profissional de saúde de nível técnico, para interagir por chamada de vídeo com um médico especialista localizado em um Centro de Saúde Especializada (Policlínica). As consultas presenciais são realizadas diretamente na Policlínica.

O objetivo deste artigo é apresentar o resultado da análise dos custos das duas modalidades de consulta, remota e presencial, por meio da metodologia de microcusteio *Time-Driven Activity-Based Costing* (TDABC)^15,16^. Trabalhamos com a hipótese de que as duas modalidades de consulta são não-inferiores, hipótese do ECR ancorada em outros trabalhos^8,9^.

Esta análise foi realizada a partir da perspectiva dos custos para o sistema de saúde (SUS) e para o paciente, considerando diferentes arranjos referentes aos custos de deslocamento. A comparação dos custos das diferentes modalidades de consulta pretende subsidiar com evidências, posteriores análises da capacidade produtiva das UBS e sua capacidade de absorção da teleconsulta dentro do modelo de financiamento do SUS.

## MÉTODOS

### Desenho do Estudo

Trata-se de uma análise de microcusteio que fornece informação sobre o custo de uma consulta especializada nas modalidades remota e presencial, com um médico endocrinologista, para o tratamento de diabetes tipo 2, considerando a não-inferioridade da modalidade de teleconsulta. As análises advêm de pacientes recrutados em ensaio clínico randomizado, fase II, pragmático e de não inferioridade, cujo protocolo já foi publicado anteriormente^14^.

A metodologia utilizada para o cálculo dos custos sob a perspectiva do sistema de saúde é a de custos baseados em tempo e atividade (TDABC)^15^, adotada por permitir a identificação do custo ao nível unitário do serviço dentro das condições de eficiência previstas, atuando também como métrica de comparação. Esta metodologia já vem sendo aplicada para a mensuração de custos em diversos serviços de saúde^17^e, inclusive, na de serviços de telemedicina em âmbito nacional^21^.

O TDABC parte do mapeamento das atividades envolvidas no processo de interesse e dos recursos envolvidos em cada uma delas. Em seguida, estima-se a taxa *de custo de capacidade* dos recursos, determinada pela equação:


To=Ccf/Cp

Na qual: *Tc* = taxa de custo de capacidade; *Ccf* = custo da capacidade fornecida; *Cp* = capacidade prática do recurso fornecido.

O *Ccf* é o custo de determinado recurso para a realização da atividade e a *Cp* é a quantidade de minutos dispendidos para a realização desta atividade. De outro modo, esta taxa pode ser interpretada como o custo por unidade de tempo (minuto) dos recursos envolvidos em determinada atividade^15^. No modelo TDABC, a capacidade prática é considerada como de 80% a 85% da capacidade teórica, assumindo que aproximadamente 20% do tempo de trabalho costuma ser dispendido em intervalos e 15% do tempo de uso dos equipamentos é gasto em manutenção e reparos^16^.

Para este cálculo na perspectiva do paciente, foram estimados os custos relacionados ao deslocamento (custos de transporte) para a Policlínica e para a UBS de referência. Os dados foram coletados por meio de um questionário preenchido pelos pacientes após a consulta, com perguntas sobre o tipo de transporte utilizado e o tempo de percurso para cada uma das localidades (UBS e Policlínica).

Finalmente, para a extrapolação da análise para outros municípios, foram utilizados dados referentes a fonte de financiamento para o transporte de pacientes segundo a Portaria SAS nº 055/1999 – Tratamento Fora do Domicílio (TFD)^22^–, que garante transporte e hospedagem para tratamento. Segundo os dados da tabela TFD (Portaria nº 2.488/2007), para os casos nos quais o município não fornece o transporte, o paciente recebe uma ajuda de custo de R$8,40 para alimentação sem pernoite e R$4,75 para cada 50km de deslocamento terrestre.

### Amostra

A amostra foi composta por 246 pacientes, que foram inseridos no estudo randomizado respeitando critérios de inclusão pré-estabelecidos^14^. As 246 consultas foram realizadas entre janeiro de 2021 e janeiro de 2023 e referem-se à primeira consulta dos pacientes com as médicas especialistas, sendo 123 consultas de cada modalidade.

As estatísticas descritivas referentes ao perfil dos participantes, com relação ao sexo e faixa etária, são apresentadas na Tabela 1.

**Tabela 1:** Estatísticas descritivas do perfil dos participantes das duas modalidades de consulta.

Modalidade consulta	% Masculino	% Feminino	Média idade (anos)	Desvio padrão (idade)
Presencial	39	61	60,12	9,83
Teleconsulta	42	58	60,23	11,04

Fonte: dados do Projeto Teleconsulta

### Fonte de Dados

O cálculo do salário/mês médio dos médicos, técnicos de enfermagem e da equipe administrativa das UBS e da Policlínica foi feito a partir dos dados disponíveis no Portal da Transparência do Município de Joinville^23^.

Os dados relativos ao custo de materiais e itens consumíveis foram disponibilizados pela Secretaria Municipal de Saúde de Joinville para três UBS e usados como referência para o custo de materiais e consumíveis das demais unidades e da Policlínica.

Para os custos do equipamento necessário para a teleconsulta (computador, plataforma de videoconferência, assinatura digital), foram utilizados os valores gastos no âmbito do Projeto Teleconsulta, considerando uma vida útil de 5 anos (taxa de depreciação de 20%) e uma taxa de desconto intertemporal de 4,5% ao ano (índice de inflação IPCA-IBGE médio de 2020).

O tempo relativo aos procedimentos pré-consulta (registro dos pacientes e triagem) foi coletado pelo pesquisador de campo do projeto e a duração das consultas foi registrada pelas médicas endocrinologistas responsáveis, considerando o início e o fim da consulta.

Para a análise dos custos de transporte, foram atribuídos valores referentes ao valor da passagem de ônibus, uso de combustível e de viagens de táxi ou veículos de aplicativo no ano de 2020 (ano para o qual todos os cálculos estão sendo realizados), a partir dos dados coletados no questionário respondido pelos pacientes após a consulta. Um total de 135 pacientes preencheram todo o questionário, sendo que todos forneceram dados de deslocamento para a UBS mais próxima e 93 para a Policlínica (42 participantes nunca tinham passado pela instituição).

### Análise dos Dados

O mapeamento das atividades que fazem parte das duas modalidades de consulta foi realizado a partir do fluxograma previsto no Projeto Teleconsulta^14^. Como mostra o Quadro, as atividades das duas modalidades de consulta e os profissionais envolvidos são os mesmos, com exceção do teste de equipamento e de seu uso, no caso da consulta remota.

**Quadro:** Atividades envolvidas no processo da consulta presencial e da teleconsulta.

Teleconsulta		Consulta presencial	
Subatividade	Responsável	Subatividade	Responsável
Recepção e registro dos pacientes	Administrativo UBS	Recepção dos pacientes	Administrativo Policlínica
Realização de exames preliminares (sinais vitais)	Técnico de enfermagem UBS	Realização de exames preliminares (sinais vitais)	Técnico de enfermagem Policlínica
Teste do equipamento	Técnico de enfermagem UBS		
Teleconsulta	Médico especialista	Consulta	Médico especialista

UBS: unidade básica de saúde.

Para o cálculo dos custos de transporte, foram considerados dados relativos ao tempo médio de percurso para cada unidade de saúde e o custo relativo de cada meio de transporte, ponderados pela proporção de pacientes que declaram fazer uso deles.

As análises se concentraram em uma descrição dos custos e duração das diferentes etapas das consultas, com informações de custos médio e comparações percentuais.

A avaliação da diferença entre médias de tempo de duração de cada modalidade de consulta foi feita por meio do teste *t-student* no software R.

## RESULTADOS

A capacidade prática considerada para os técnicos de enfermagem e pessoal administrativo foi 176 horas, ou 10.560 minutos (80% de 220 horas/mês). Para o caso das médicas endocrinologistas, a capacidade prática considerada foi 96 horas, ou 5.760 minutos (80% de 120 horas/mês).

Para a categoria de materiais e itens consumíveis, considerou-se 85% da sua capacidade teórica, segundo o modelo TDABC, resultando em 36.720 minutos/mês de capacidade prática e, para os equipamentos necessários para a teleconsulta, a capacidade prática considerada corresponde à carga horária dos médicos (96 horas/mês), segundo hipótese que estes equipamentos serão utilizados exclusivamente para a realização de teleconsultas. Considera-se aqui o valor para uma UBS.

A partir destes dados foi calculada a taxa de custo de capacidade, ou custo por minuto, expostos na Tabela 2 onde é possível observar que, para o caso de Joinville, o custo médio relativo ao pessoal administrativo na UBS é maior do que o observado na Policlínica. Para o caso dos técnicos de enfermagem, não há diferença significativa.

**Tabela 2:** Taxa de custo de capacidade: custo por minuto dos recursos.

Atividades	Horas/mês	Minutos/mês	Salário médio/custo/mês	Desvio padrão (R$)	Taxa de custo de capacidade (R$)
Técnico enfermagem	176	10.560	5.346,48	1.425,06	0,51
Média-UBS					
Técnico enfermagem	176	10.560	5.408,42	1.871,02	0,51
Média-Policlínica					
Pessoal administrativo	176	10.560	4.546,22	1.756,42	0,43
Média-UBS					
Pessoal administrativo	176	10.560	3.060,78	927,11	0,29
Média-Policlínica					
Médicas	96	5.760	10.584,00	2.049,14	1,84
Materiais e consumíveis	612	36.720	2.547,31	NA	0,07
Equipamento teleconsulta	96	5.760	288,10	NA	0,05

Fonte: dados do Portal da Transparência de Joinville e dados do Projeto Teleconsulta.

UBS: unidade básica de saúde; NA: não aplicável.

Na segunda etapa, foi coletado o tempo de duração exigido para concluir cada atividade. Nas consultas presenciais a média de duração do registro dos pacientes foi de 9,6 minutos e o tempo de realização dos exames preliminares de 5,3 minutos e, na teleconsulta, de 10,5 e 4,7, respectivamente.

Na média, as consultas têm 42,8 minutos de duração, sendo a teleconsulta menos de 1 minuto (48 segundos) mais curta do que a da consulta presencial. O desvio padrão também foi menor na modalidade remota: 8,8 minutos ante a 9,6 minutos na presencial.

Considerando um nível de significância de 95%, não rejeitamos a hipótese nula, de que as médias de duração das etapas descritas são iguais estatisticamente. Assim, o tempo de duração considerado para a estimação dos custos pelo modelo TDABC de ambas as modalidades de consulta foi de: 5 minutos para registro dos pacientes, 10 minutos para a realização exames preliminares e 42,8 minutos para realização das consultas.

### Custo sob a Perspectiva do SUS: Cálculo do Custo Contábil

Após a obtenção dos custos dos recursos utilizados ao longo do processo, estes foram associados às respectivas atividades e ao seu tempo médio de duração, resultando no custo contábil de R$ 92,4 para uma teleconsulta, valor 4,5% maior do que o da consulta presencial, que ficou em R$ 88,4, como mostra a Tabela 3. É evidenciado, também, que a etapa da realização da consulta é responsável por mais de 90% dos custos, em função do valor do salário das médicas em relação aos outros custos.

**Tabela 3:** Custo por atividades nas diferentes modalidades de consulta.

Subatividade	Responsável	Teleconsulta					Consulta presencial				
		Custo/min (R$)		Tempo (min)	Custo (R$)	% do custo de cada etapa	Custo/min (R$)		Tempo (min)	Custo (R$)	% do custo de cada etapa
		Salários (R$)	Estrutura e materiais				Salários (R$)	Estrutura e materiais			
Registro do paciente	Pessoal administrativo	0,43	R$ 0,07	10	5	5	0,29	R$ 0,07	10	3,6	4
Exames preliminares (sinais vitais)	Técnico enfermagem	0,51	R$ 0,07	5	2,9	3	0,51	R$ 0,07	5	2,9	3
Teste do equipamento	Técnico enfermagem	0,51	R$ 0,11	1	0,62	1					
Teleconsulta	Médico especialista	1,84	R$ 0,12	42,8	83,9	91	1,84	R$ 0,07	42,8	81,9	93
Total					92,41	100				88,42	100

O valor adicional da teleconsulta em relação à presencial é resultado do valor do equipamento para a realização da consulta e do salário mais alto do pessoal administrativo nas UBS.

Neste cenário, a diferença de custos entre as modalidades é bastante sensível a variações na duração da consulta. Por exemplo, para uma situação em que, na média, o tempo de duração da teleconsulta é um minuto mais curto do que o da consulta presencial, o custo contábil das duas modalidades seria igual.

Esta estimativa de custo unitário por consulta parte do pressuposto que todo o período de capacidade prática das UBS seja utilizado para realização de teleconsultas, ou, um atendimento médio de 135 consultas/mês por UBS.

### A Perspectiva do Paciente e os Custos de Deslocamento

Para além do custo contábil, examinamos a perspectiva dos pacientes, incorporando as estimativas de custos relacionados ao deslocamento deles em cada modalidade de consulta nas análises.

A Tabela 4 apresenta o tempo e o custo médio da viagem para cada localidade, considerando os percursos de ida e volta.

**Tabela 4:** Custo médio de deslocamento para a UBS e Policlínica (ida e volta).

Deslocamento	Policlínica Boa Vista						UBS						Dif. tempo médio de percurso Policlínica-UBS (min)
	n	%	Tempo médio de percurso (min)	Desvio padrão (min)	Custo percurso (R$)	Custo ponderado (R$)	n	%	Tempo médio de percurso (min)	Desvio padrão (min)	Custo percurso (R$)	Custo ponderado (R$)	
Carro	55	59	48	37	12	7,1	70	52	24,6	27,8	6,2	3,2	23,4
Táxi/aplicativo	17	18	34,4	38,7	44	8	10	7	20,4	31,5	26,1	1,9	14
Ônibus	18	19	67,5	40	9,5	1,8	4	3	20,5	38,9	9,5	0,3	47
Bicicleta/a pé	3	3	25	35,3	0	0	51	38	22,5	33,2	0	0	2,5
Total	93	100	48,5			17	135	100	23,4		41,8	5,4	25,2

UBS: unidade básica de saúde.

Em Joinville, uma cidade de porte médio com aproximadamente 600 mil habitantes, o custo médio com transporte para a realização de uma consulta na Policlínica (R$ 17,00) é mais de três vezes maior do que para a realização de uma consulta na UBS do bairro (R$ 5,40).

Com relação ao tempo de percurso, o tempo de deslocamento até a Policlínica é, em média, 25 minutos maior do que para a UBS mais próxima. A maior diferença está no deslocamento de ônibus, em que o tempo de percurso relatado é 47 minutos maior para a Policlínica. Com efeito, apenas 3% da amostra declarou usar esse meio de transporte para ir até a UBS de seu bairro, para onde, devido à proximidade, 38% declarou ir a pé ou de bicicleta.

Adicionando o custo sob a perspectiva do paciente ao cálculo do custo contábil, obtemos um cenário no qual o custo da consulta presencial é 7,7% maior que o da teleconsulta:


$$\begin{array}{cc} & C_{\text{uoto }}\text{ TELE }=92,4+5,4=97,8\\ & C_{\text{P }}\;\text{ PRESENCIAL }=88,4+17=105,4 \end{array}$$

### Estimação dos Custos de Transporte para Pacientes de outros Municípios

Nesta estimação, trabalhamos com um cenário no qual o tratamento de diabetes pelo SUS inicia-se na atenção primária da cidade de origem do usuário e é encaminhado para outras localidades que possuam serviços complementares de atenção especializada. Nestes casos, é obrigação do município de origem transportar ou arcar com os custos de transporte dos pacientes e mesmo dos acompanhantes para tratamento fora do seu domicílio. No caso de consultas especializadas de endocrinologia, o município de Joinville é referência para seis outros municípios (Araquari, Barra do Sul, Garuva, Itapoá, São Francisco do Sul) da região^a^. Ou seja, todos esses municípios têm de arcar com os custos de transporte e alimentação dos pacientes que se deslocam para Joinville para consulta especializada com um endocrinologista.

Considerando que o custo para a realização da teleconsulta nas UBSs desses municípios é equivalente ao calculado e utilizando os valores do TFD para um município a 50 km de Joinville, para o caso da consulta presencial há um adicional de R$ 17,90, que inclui o transporte e alimentação do paciente, sem acompanhante.


$$\begin{array}{cc} & C_{\text{usto }}\text{ TELE }=92,4\\ & C_{\text{uto }}\text{ PRESENCIAL }=88,4+17,9=106,3 \end{array}$$

Neste cenário, considerando apenas o custo contábil sob a perspectiva do SUS, a consulta presencial seria 15% mais cara do que a teleconsulta realizada em uma UBS local.

Ainda neste caso, podemos analisar a perspectiva dos diferentes gestores: o do município de residência do paciente e o do município de realização da consulta, no caso, Joinville. Dentro do sistema de financiamento do SUS para os fundos municipais, o valor da consulta seria de Joinville, portanto, faz sentido comparar, na perspectiva do gestor do município de residência do paciente, os custos de transporte com os custos relativos à realização da teleconsulta na UBS local. Neste caso, a partir dos valores expostos na tabela 3, teríamos que o custo para o gestor local seria de R$ 10,66, ou seja, 40% menor do que o custo de transporte calculado a partir dos dados do TFD.

## DISCUSSÃO

Nesta análise, os custos da teleconsulta se mostraram levemente maiores do que da modalidade presencial, mas bastante sensíveis a variação de parâmetros, como a duração da consulta. Quando a perspectiva do paciente é considerada, ou em um cenário no qual o paciente tem de se deslocar para outro município para a realização da consulta, a modalidade remota apresenta um menor custo. O custo de oportunidade relacionado ao tempo “economizado” pelos pacientes na modalidade remota de consulta não foi considerado, mas em grandes centros urbanos e em deslocamentos intermunicipais pode ser bastante relevante.

No caso de consultas realizadas em outros municípios, a gestão e o custo dos transportes varia muito, mas pode ser um montante significativo. Em estudo^24^ realizado no município de Santa Maria (RS), o custo médio por paciente transportado foi de R$ 32,94, mais um adicional de R$2,40 por quilometro rodado. Outro estudo^25^, em Victor Meireles (SC), que possui apenas 5 mil habitantes, encontrou um gasto médio mensal de R$ 37.835,16 com transporte de pacientes.

Outros aspectos econômicos poderiam sofrer impacto pela implantação da teleconsulta, como o absenteísmo de usuários em consultas e exames, o que gera grande desperdício de recursos. No Brasil, estudos apontam taxas superiores a 25% de absenteísmo na atenção especializada ^26,27^, que tendem a ser maiores do que as registradas na atenção primária^27,28^. Considerando que a distância até o local de atendimento está inversamente relacionada à taxa de absenteísmo^29^, a possibilidade de realizar o atendimento especializado mais próximo da residência, ou mesmo na residência dos pacientes, poderia proporcionar um uso mais eficiente dos recursos através de uma possível diminuição das faltas nas consultas.

Evidências de outros países também destacam o potencial econômico da teleconsulta. Em uma revisão sistemática^30^ de estudos conduzidos em países de alta renda usando anos de vida ajustados pela qualidade, os resultados apontam que a telemedicina é custo-efetiva e, no contexto de países de baixa renda, outro estudo com dados de mais de 25 mil teleconsultas, os resultados sugerem benefícios econômicos consideráveis e grandes potencial da telemedicina para melhorar o tratamento de doenças crônicas em áreas de pouco recurso^31^.

Neste sentido, a disponibilidade e a qualidade dos recursos disponíveis pode ser um obstáculo para a implantação da teleconsulta e a efetivação dos benefícios a ela associados. No modelo aqui tratado, onde as teleconsultas são realizadas nas UBS, as unidades já contavam com itens como conexão e sala para realização da teleconsulta, mas um estudo recente^32^aponta que a maioria das UBS não apresenta estrutura mínima para a realização de uma teleconsulta, com desigualdades regionais e de porte populacional evidentes e maiores carências nas regiões que mais poderiam se beneficiar de tal serviço, como o Norte e Nordeste.

Além da infraestrutura, a baixa remuneração das consultas e a desigualdade na distribuição de médicos pelo território são alguns dos fatores que contribuem para que a oferta de cuidado especializado seja um dos grandes gargalos do sistema^33,34^, onde a telemedicina tem potencial para atuar como um atenuante, se combinada com a oferta de serviços de forma hierarquizada e regionalizada, garantindo uma economia de escala adequada. Os resultados do presente estudo contribuem com o corpo de evidencias sobre o tema em âmbito nacional, mas apresentam algumas limitações. Os valores absolutos estimados não correspondem exatamente aos valores de uma consulta “real”, pois foram calculados no âmbito de um estudo específico. Porém, os valores relativos à diferença entre o custo das duas modalidades de consulta, em conjunto com outros aspectos aqui tratados, oferecem estimativas do impacto econômico da implementação desta tecnologia, que devem ser também examinadas em relação ao nível de necessidade da população e aos efeitos subsequentes sobre o acesso a saúde^3^, especialmente em um sistema público e universal.

Por ser um estudo regional (Joinville/SC), os valores de remuneração de pessoal podem divergir de demais localidades brasileiras. Ademais, a validade externa dos dados, considerando a implementação da teleconsulta em outros cenários que não o da endocrinologia, e mais especificamente do diabetes, é baixa.

Novos estudos complementando a temática desta avaliação, feitos de forma multicêntrica e com outras especialidades médicas, podem acrescentar maior robustez à presente análise e para identificação de potencialidades e limites da telemedicina no Brasil.

## CONCLUSÃO

Nossos achados demonstraram que os custos para a realização da teleconsulta em uma UBS se mostrou 4,5% maior do que para uma consulta presencial realizada em um centro especializado. A duração das duas modalidades de consulta se mostram estatisticamente iguais, contudo, com a remuneração das médicas sendo responsável por mais de 90% dos custos, as estimações são bastantes sensíveis à variação do tempo de duração da consulta: mantidos todos os outros parâmetros constantes, se a teleconsulta tivesse um minuto a menos de duração o custo das duas modalidades de consulta seria igual.

Quando considerados os custos de transporte para o paciente e para casos nos quais o município é o responsável pelos custos de deslocamento, o valor da consulta presencial passa a ser, respectivamente, 7,7% e 15% maior do que o da modalidade remota.

## Footnotes

a Informação fornecida pela Secretaria Municipal de Saúde de Joinville.
